# Vitamin B6 rescues insulin resistance and glucose‐induced DNA damage caused by reduced activity of *Drosophila* PI3K

**DOI:** 10.1002/jcp.30812

**Published:** 2022-06-09

**Authors:** Elisa Mascolo, Francesco Liguori, Chiara Merigliano, Ludovica Schiano, Eleonora Gnocchini, Eleonora Pilesi, Cinzia Volonté, Martino L. Di Salvo, Roberto Contestabile, Angela Tramonti, Fiammetta Vernì

**Affiliations:** ^1^ Department of Biology and Biotechnology “Charles Darwin” Sapienza University of Rome Rome Italy; ^2^ Preclinical Neuroscience, IRCCS Santa Lucia Foundation Rome Italy; ^3^ Department of Molecular and Computational Biology University of Southern California Los Angeles California USA; ^4^ Istituto Pasteur Italia ‐ Fondazione Cenci Bolognetti and Department of Biochemical Sciences “A. Rossi Fanelli” Sapienza University of Rome Rome Italy; ^5^ Institute for Systems Analysis and Computer Science “A. Ruberti”, National Research Council (IASI—CNR) Rome Italy; ^6^ Institute of Molecular Biology and Pathology, National Research Council (IBPM‐CNR) Rome Italy

**Keywords:** DNA damage, *Drosophila*, insulin signaling, phosphatidylinositol 3‐Kinase, pyridoxal phosphate

## Abstract

The insulin signaling pathway controls cell growth and metabolism, thus its deregulation is associated with both cancer and diabetes. Phosphatidylinositol 3‐kinase (PI3K) contributes to the cascade of phosphorylation events occurring in the insulin pathway by activating the protein kinase B (PKB/AKT), which phosphorylates several substrates, including those involved in glucose uptake and storage. PI3K inactivating mutations are associated with insulin resistance while activating mutations are identified in human cancers. Here we show that RNAi‐induced depletion of the *Drosophila* PI3K catalytic subunit (Dp110) results in diabetic phenotypes such as hyperglycemia, body size reduction, and decreased glycogen content. Interestingly, we found that hyperglycemia produces chromosome aberrations (CABs) triggered by the accumulation of advanced glycation end‐products and reactive oxygen species. Rearing *PI3K* ^*RNAi*^ flies in a medium supplemented with pyridoxal 5′‐phosphate (PLP; the catalytically active form of vitamin B6) rescues DNA damage while, in contrast, treating *PI3K* ^*RNAi*^ larvae with the PLP inhibitor 4‐deoxypyridoxine strongly enhances CAB frequency. Interestingly, PLP supplementation rescues also diabetic phenotypes. Taken together, our results provide a strong link between impaired PI3K activity and genomic instability, a crucial relationship that needs to be monitored not only in diabetes due to impaired insulin signaling but also in cancer therapies based on PI3K inhibitors. In addition, our findings confirm the notion that vitamin B6 is a good natural remedy to counteract insulin resistance and its complications.

## Graphical abstract

RNAi‐induced depletion of the *Drosophila* phosphatidylinositol 3‐kinase (PI3K) catalytic subunit results in diabetic phenotypes such as hyperglycemia, body size reduction, and impaired glycogen synthesis. We found that hyperglycemia produces chromosome aberrations (CABs) triggered by the accumulation of advanced glycation end‐products and reactive oxygen species. Vitamin B6 treatment rescues DNA damage and diabetic phenotypes, while its inhibitor 4‐deoxypyridoxine strongly enhances CAB frequency. Our results provide a strong link between impaired PI3K activity and genomic instability and confirm that vitamin B6 is a good remedy to counteract insulin resistance and its complications.

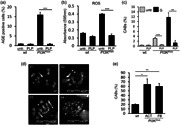

**Abbreviations**
4DP: 4‐deoxypyridoxine
AA: ascorbic acid
AGEs: advanced glycation end‐products
ALA: α‐lipoic acid
CABs: chromosome aberrations
NBT: nitro blue tetrazolium
PI3K: phosphatidylinositol 3‐kinase
PLP: pyridoxal 5′‐phosphate
ROS: reactive oxygen species
T2D: type 2 diabetes

## 1 INTRODUCTION

Insulin signaling is a complex pathway that, besides controlling cell growth, guarantees a correct balance between glucose uptake and storage, thus contributing to glucose homeostasis maintenance (Lizcano & Alessi, 2002).

Impaired signaling results in insulin resistance—a condition that preludes type 2 diabetes (T2D)—in which normal amounts of insulin are inadequate to stimulate insulin response from fat, muscle, and liver cells (Muoio & Newgard, 2008). The interaction between insulin and its receptor on the surface of target cells leads to the phosphorylation of the insulin receptor substrate protein, which in turn, recruits phosphatidylinositol 3‐kinase (PI3K) to the membrane. This kinase phosphorylates phosphatidylinositol‐(4,5)‐bisphosphate (PIP2) generating phosphatidylinositol‐(3,4,5)‐trisphosphate (PIP3). Then, PIP3 recruits the PH domain‐containing protein kinase B (PKB/AKT) to the plasma membrane and permits its further activation by phosphoinositide‐dependent kinase 1 (PDK1). Activated AKT inhibits the transcription factors of the FOXO (Forkhead box, subgroup “O”) family and phosphorylates several downstream targets including those responsible for the glucose metabolism regulation (Lizcano & Alessi, 2002).

PI3K belongs to a family of evolutionarily conserved proteins grouped into three classes (I–III), each playing distinct roles in cellular signal transduction pathways. Class IA PI3Ks are involved in insulin signaling and consist of heterodimers made of a p85 regulatory subunit and a p110 catalytic subunit. Three genes—*PIK3R1*, *PIK3R2*, and *PIK3R3*—encode the p85α, p85β, and p55γ isoforms of the p85 regulatory subunit. Other three genes—*PIK3CA*, *PIK3CB*, and *PIK3CD*—encode the p110 catalytic subunit isoforms p110α, p110β, and p110δ (Engelman et al., 2006). The p85 regulatory subunit mediates the activation of IA PI3Ks by its interaction with receptor tyrosine kinases. This binding relieves the basal inhibition of p110 by p85 and allows the binding of the p85–p110 heterodimer to its substrate (PIP2) at the plasma membrane.

Heterozygous mutations in *PIK3R1* have been associated with the SHORT syndrome, a disorder characterized by short stature, partial lipodystrophy, and insulin resistance (Chudasama et al., 2013). The gene encoding for p110α, namely, *PIK3CA*, is mutated or amplified in several solid and hematological cancers (Alqahtani et al., 2019). *PIK3CA* mutations are clustered in hot spots and induce a gain of function, upregulating the activity of the mutant p110α (Zhao & Vogt, 2008). Based on these studies, targeting PI3K has been looked at as a promising cancer therapy. Several PI3K inhibitors have gone through clinical trials, but, despite the therapeutic benefit, their administration was associated with some unusual toxicities, due to PI3K inhibition in nonmalignant cells (Yang et al., 2019). It is therefore essential to better characterize the effects of PI3K inactivation and to develop dietary or pharmaceutical approaches able to improve the efficacy/toxicity ratio of PI3K inhibitors.

In *Drosophila*, the insulin signaling pathway is similar to the human counterpart (Liguori et al., 2021) Class I PI3Ks are heterodimers composed of the catalytic subunit PI3K92E (also named Dp110), and the regulatory subunit Dp60 encoded by the gene *PI3K21B* (Weinkove et al., 1997). Mutations in Dp110 and Dp60 are lethal and result in the arrest or delay of larval growth (Weinkove et al., 1999). Different allelic combinations or RNAi‐mediated depletion of the catalytic subunit Dp110 resulted in flies with small body sizes and an increased glucose concentration in larval hemolymph (Murillo‐Maldonado et al., 2011; Ugrankar et al., 2015).

Growing evidence indicates that hyperglycemia causes oxidative stress because of an imbalance between free radical formation and their control by natural antioxidants, thus leading to the development of micro‐ and macrovascular diabetes complications. Consistently, chromosome aberrations (CABs) and micronuclei are found in diabetic patients (da Silva et al., 2013; Franzke et al., 2020). Several vitamins, with antioxidant properties and/or working as coenzymes in reactions involved in DNA metabolism, exert a protective role against diabetes complications (Deshmukh et al., 2020). In particular, the active form of vitamin B6, pyridoxal 5′‐phosphate (PLP), prevents retinopathies and nephropathies (Ellis et al., 1991; Nix et al., 2015) and, in general, improves the metabolic control in both diabetic patients and animal models (Mascolo & Vernì, 2020).

In this study, we found that the silencing of the *PI3K92E* gene, encoding the catalytic subunit (Dp110) of *Drosophila* PI3K, results in diabetic phenotypes, such as increased glucose content in the hemolymph, decreased glycogen content, and reduced body size. In addition, we found that Dp110 depletion caused CABs triggered by hyperglycemia. Interestingly, vitamin B6 supplementation was able to rescue both CABs and diabetic phenotypes in flies carrying a *PI3K92E^RNAi^* construct. The implications of these results are discussed.

## 2 MATERIALS AND METHODS

### 2.1 Drosophila stocks

*PI3K92E_v107390* and *PI3K92E_v38985* lines were obtained from Vienna *Drosophila* Resource Center. *ppl‐Gal4* (BL58768) and *elav‐Gal4* (BL25750) driver lines were obtained from the Bloomington *Drosophila* Stock Center. Oregon R strain was used as a wild‐type control. All stocks were maintained and crossed at 25°C on a standard cornmeal agar‐yeast‐sugar medium or on a standard medium supplemented with 1 mM PLP. The used balancers and genetic markers are described in detail on FlyBase (http://flybase.bio.indiana.edu/).

### 2.2 Chromosome cytology and immunostaining

Colchicine‐treated *Drosophila* chromosome preparations were obtained as previously described (Gatti & Goldberg, 1991). For advanced glycation end‐product (AGE) detection, brain preparations from third instar larvae were carried out according to (Marzio et al., 2014) and stained with a rabbit anti‐human AGE primary antibody (1:200; ab23722; Abcam) and with the anti‐rabbit Alexa Fluor 555‐conjugated secondary antibody (1:300; Molecular Probes).

One percent glucose, 10 mM α‐lipoic acid (ALA), or 40 mM ascorbic acid (AA) brain treatments for CAB or AGE detection were performed as previously described (Marzio et al., 2014).

To evaluate the effects of the 4‐deoxypyridoxine (4DP) on CABs, 2 mM 4DP was added to the growth medium. Glucose, ALA, and 4DP concentrations were chosen according to (Marzio et al., 2014), while AA was concentrated according to (Vaccaro et al., 2020).

All fixed preparations were mounted in Vectashield H‐1200 with 4,6‐diamidino‐2‐phenylindole (Vector Laboratories) for DNA staining. All cytological preparations were examined with a Carl Zeiss Axioplan fluorescence microscope equipped with an HBO100W mercury lamp and a cooled charged‐coupled device (CCD camera; Teledyne Photometrics).

### 2.3 Glucose measurement, glycogen quantification, and weight analysis

For glucose measurement, hemolymph was collected from third instar larvae as described in (Tennessen et al., 2014). Glucose concentration was measured using the GAHK20 Sigma reagent. Glycogen quantification was performed on whole larval body extracts (Tennessen et al., 2014). Glycogen was digested into glucose monomers by amyloglucosidase (Sigma A1602), then its concentration was quantified by subtracting the glucose absorbance from the total glycogen + glucose absorbance and normalized to total protein content, quantified by Bradford assay (Post et al., 2018).

For weight analysis, five to six samples of 15 flies each were weighted with a precision weight scale (Gibertini E42; range: 0.1 mg–120 g). Flies were reared under the same growth conditions and were age‐matched (2 days old) before weighing.

### 2.4 Nitro blue tetrazolium assay

Reactive oxygen species (ROS) measurement was performed in larval hemolymph from larvae reared in standard, PLP‐ or AA‐supplemented medium, using the nitro blue tetrazolium (NBT) as previously described (Mascolo et al., 2021).

### 2.5 Statistical analysis

Results are expressed as mean ± *SEM*; *p* < 0.05 were considered statistically significant. Statistical analysis of the data were performed with the two‐tailed Student's *t*‐test.

## 3 RESULTS

### 3.1 The depletion of the PI3K catalytic subunit (Dp110) causes diabetes in flies

**Figure 1:**
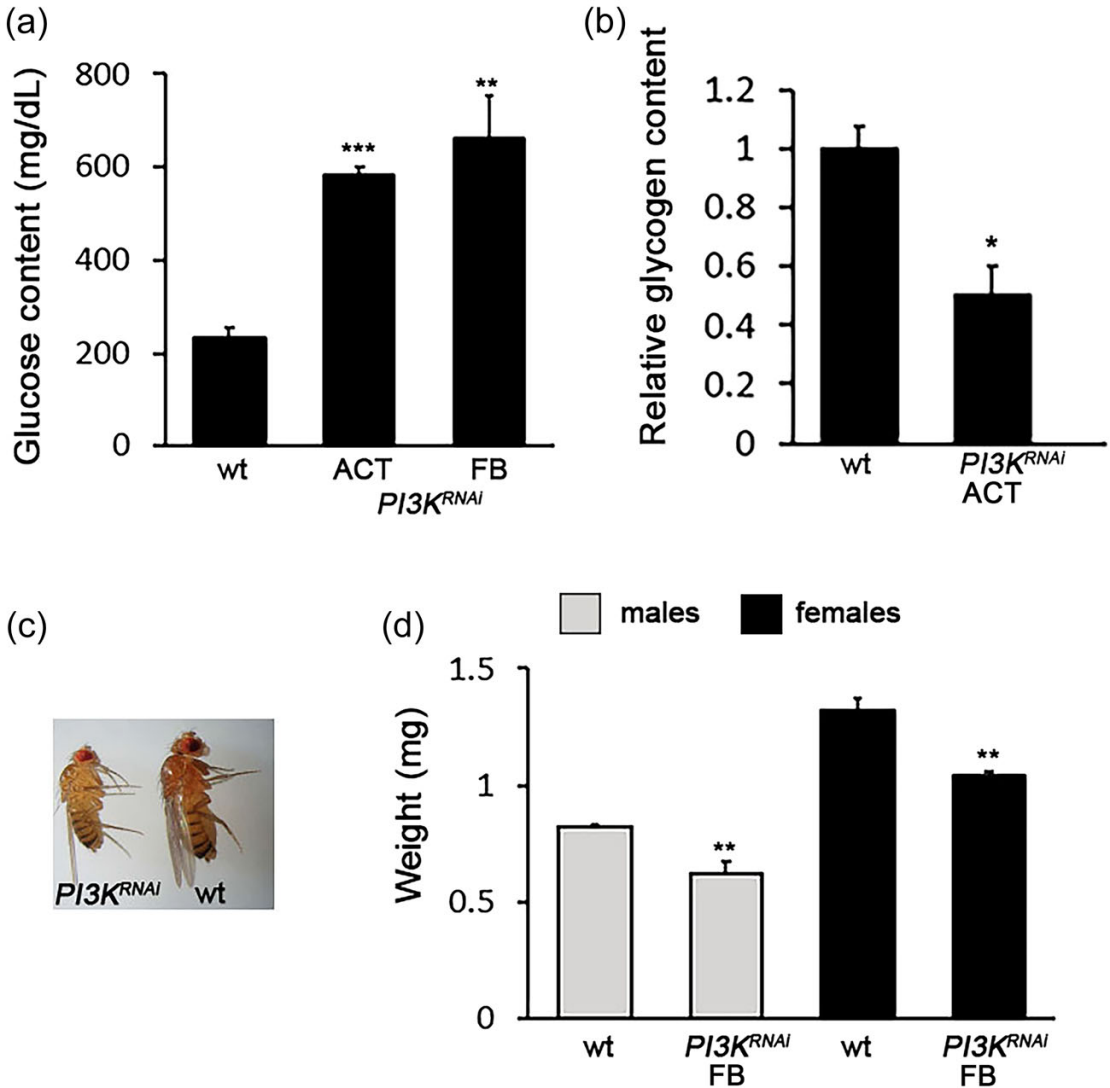
The silencing of *PI3K92E* results in diabetic phenotypes. (a) Glucose content in larval hemolymph in wild‐type and RNAi individuals in which the *PI3K92E* gene was ubiquitously silenced or specifically silenced in the fat body. Columns are the mean of five independent sample measurements ±SEM. (b) Relative glycogen content in *PI3K* ^*RNAi*^ larvae. Columns are the mean of three independent biological replicates ±*SEM*. (c) Silencing of *PI3K92E* in the fat body results in reduced body size. (d) Weight measurement. Each column represents the mean weight (±*SEM*) of single flies obtained from three independent experiments. ACT, ubiquitous driver; FB, fat body driver; wt, wild‐type. Significant difference in the Student's *t*‐test with *p < 0.05, **p < 0.01, and ***p < 0.001, respectively.

To investigate the spectrum of the phenotypes caused by depletion of the *Drosophila* PI3K catalytic subunit (Dp110), we silenced the *PI3K92E* gene in the *PI3K92E_v107390* line using RNA interference; individuals carrying the silenced gene are hereinafter referred to as *PI3K92E* ^*RNAi*^. Both ubiquitous (*actin‐Gal4* driver) and fat body‐specific (*ppl‐Gal4 driver*) *PI3K92E* silencing (*Drosophila* fat body corresponds to vertebrate liver and adipose tissue) increased the glucose content in larval hemolymph (Figure 1a). In addition, Dp110 depletion decreased glycogen content (Figure 1b). Ubiquitous expression of *PI3K92E* ^*RNAi*^ construct produced lethal effects on adults, while fat body‐specific silencing resulted in flies showing reduced body size, a typical diabetic trait (Figure 1c,d).

The inactivation of *PI3K92E* in another RNAi line (*PI3K92E_v38985*) yielded the same results (Figure S1a,b).

### 3.2 Dp110 depletion causes chromosome damage

**Figure 2:**
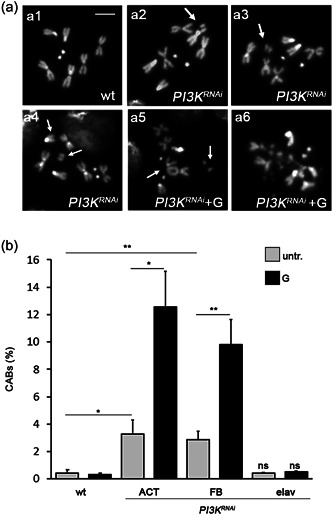
Dp110 depletion causes chromosome breaks. (a) Examples of chromosome aberrations (CABs) in *PI3K* ^*RNAi*^ untreated or 1% glucose treated neuroblasts: (a1) wild‐type female metaphase; (a2) chromatid deletion of a major autosome, arrow; (a3) isochromatid deletion of a major autosome, arrow; (a4) isochromatid deletion of the X chromosome, arrow; (a5) dicentric chromosome (autosome–autosome) accompanied by acentric fragments, arrows; (a6) metaphase with multifragmented chromosomes. Scale bar = 5 μm. (b) CAB quantification. Each column represents the mean value ± *SEM* obtained by scoring at least 1000 cells (five to six brains) for each condition. ACT, ubiquitous driver; FB, fat body driver; elav, brain driver; G, 1% glucose treatment; ns, nonsignificant; untr., untreated; wt, wild‐type. Significant difference in the Student's *t*‐test with *p < 0.05 and **p < 0.01, respectively.

Growing evidence associates chromosome damage with diabetes (Franzke et al., 2020; Tatsch et al., 2012). By supposing that increased glucose content in the hemolymph could result in enhanced glucose uptake in the brain, we tested whether Dp110 depletion may cause CABs in larval neuroblasts, the most suitable *Drosophila* cells to examine chromosomes. As shown in Figure 2, *PI3K92E* ^*RNAi*^ brains displayed 3.28% of CABs (vs. 0.5% in controls) when *PI3K92E* was ubiquitously silenced (*Act‐Gal4* driver) and 2.87% when it was specifically silenced in the fat body (*ppl‐Gal4 driver*). In addition, 1% glucose (G) treatment of isolated brains from *Act‐Gal4*>*PI3K* ^*RNAi*^ or *ppl‐Gal4*>*PI3K* ^*RNAi*^ larvae yielded 10%–12.5% of CABs; interestingly these brains displayed also 1.5%–2.3% of cells in which chromosomes appeared extensively fragmented (Figure 2a6). All these data were also confirmed using the *PI3K92E_v39885* RNAi line (Figure S1c).

Taken together, these results suggest that CABs due to Dp110 depletion are strongly sensitive to glucose; it is, therefore, reasonable to suppose that chromosome damage is a consequence of hyperglycemia. As a further confirmation of this hypothesis, brain‐specific silencing of *PI3K92E* (through the pan‐neuronal *Elav‐Gal4* driver) did not show any CAB (Figure 2b).

### 3.3 CABs are induced by hyperglycemia in Dp110‐depleted brain cells

**Figure 3:**
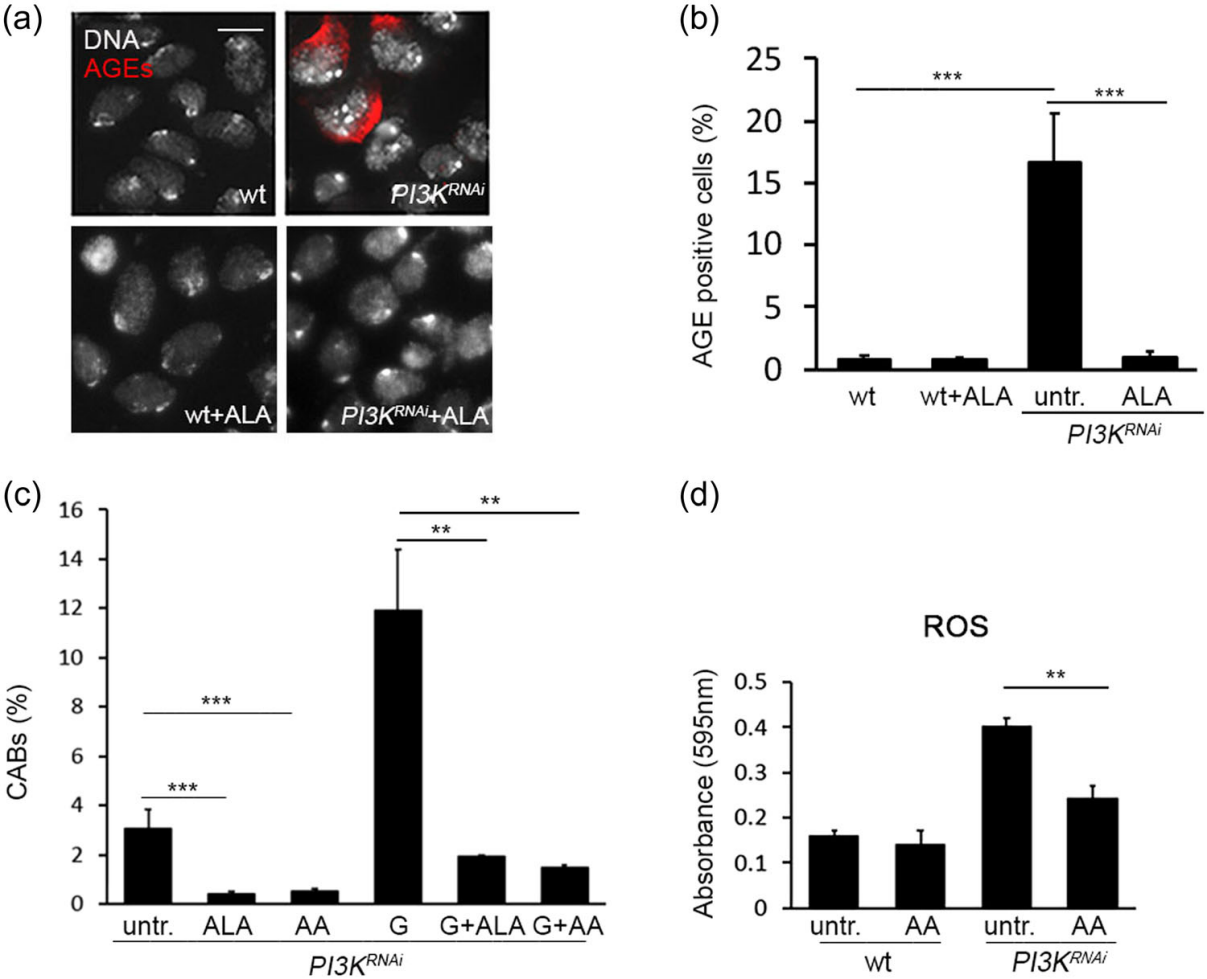
*PI3K* ^*RNAi*^ larvae accumulate advanced glycation end‐products (AGEs) and reactive oxygen species (ROS). (a) Examples of neuroblasts untreated or treated with ALA, stained with a rabbit anti‐human AGE antibody. Scale bar = 5 μm. (b) Frequencies of AGE‐positive cells in wild‐type and *PI3K* ^*RNAi*^ brains. Bars represent the mean frequencies of AGE‐positive cells ±*SEM* obtained by scoring at least 500 cells in five brains. (c) Chromosome aberration (CAB) frequency in *PI3K* ^*RNAi*^ ALA or AA‐treated neuroblasts. Each column represents the mean value ± *SEM* obtained by scoring at least 800 cells for each condition. (d) ROS quantification using nitro blue tetrazolium (NBT) assay in larval hemolymph of wild‐type and *PI3K* ^*RNAi*^ larvae untreated or AA‐treated. Columns represent a mean value ± *SEM* of three different experiments. AA, ascorbic acid; ALA, α‐lipoic acid treatment; G, 1% glucose treatment; untr., untreated; wt, wild‐type. Significant difference in the Student's *t*‐test with **p < 0.01 and ***p < 0.001, respectively.

Hyperglycemia can trigger the nonenzymatic glycation of proteins and DNA leading to the accumulation of AGEs, which are largely responsible for diabetic complications (Giacco & Brownlee, 2010). To test whether hyperglycemia induced by Dp110 depletion promoted AGE formation, we immunostained neuronal cells with an anti‐human AGE antibody. As shown in Figure 3a,b we found a significantly higher number of AGE positive cells with respect to controls (16% vs. 1%). AGE accumulation was rescued by 10 mM ALA, an antioxidant compound commonly used in managing diabetic complications (Figure 3a,b) (Golbidi et al., 2011). Interestingly, ALA also rescued the CABs resulting from Dp110 depletion, further reinforcing the hypothesis that they depend upon hyperglycemia (Figures 3c and S2). Moreover, we found that AA, another antioxidant compound, also rescued CABs in untreated and glucose‐treated brains (Figure 3c).

It is known that AGEs are accompanied by the formation of ROS, which can attack DNA causing gene and chromosome mutations (Nowotny et al., 2015). For this reason, we measured ROS in larval hemolymph by performing an NBT assay, in which the interaction of NBT with superoxide generates a product (formazan) whose absorbance directly correlates with ROS amount. As shown in Figures 3d and S1d, Dp110 depletion led to ROS accumulation, rescued by AA, further supporting the model in which CABs in Dp110‐depleted larvae are induced by high glucose levels.

### 3.4 PLP rescues the oxidative stress induced by PI3K depletion

**Figure 4:**
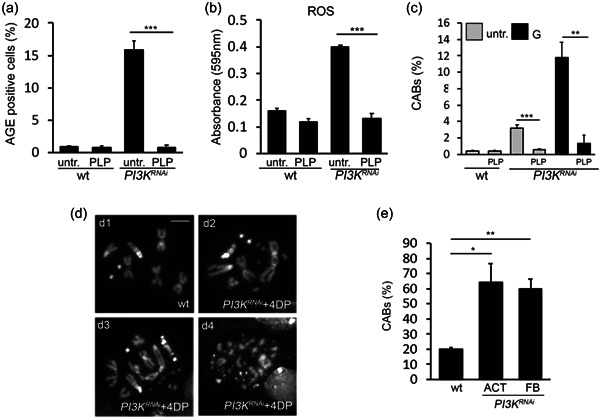
Pyridoxal 5′‐phosphate (PLP) rescues advanced glycation end‐product (AGE) accumulation and DNA damage. (a) Frequencies of AGE‐positive cells in brains from untreated and 1 mM PLP‐fed wild‐type and *PI3K* ^*RNAi*^ larvae. Bars represent the mean frequencies of AGE‐positive cells ±*SEM* obtained by examining at least 500 cells in five brains for each condition. (b) Reactive oxygen species quantification using nitro blue tetrazolium (NBT) assay in larval hemolymph of wild‐type and *PI3K* ^*RNAi*^ larvae, untreated and 1 mM PLP‐fed. Columns represent a mean value ± *SEM* of three different experiments. (c) Chromosome aberration (CAB) frequency in *PI3K* ^*RNAi*^ neuroblasts—untreated or 1% glucose treated—from larvae grown on a standard medium or 1 mM PLP supplemented medium. Each column represents the mean value ± *SEM* obtained by scoring at least 800 cells for each condition. (d) Examples of chromosome rearrangements induced by the PLP inhibitor 4‐deoxypyridoxine (4DP, 2 mM): (d1) wild‐type male metaphase; (d2) and (d3) metaphases with complex rearrangements; (d4) metaphases showing extensive chromosome fragmentation. Scale bar = 5 μm. (e) Percentage of CABs in neuroblasts from *PI3K* ^*RNAi*^ larvae grown in a 4DP‐supplemented medium. *PI3K92E* was ubiquitously silenced or specifically silenced in the fat body. Each column represents the mean value ± *SEM* obtained by scoring at least 800 cells for each condition. ACT, ubiquitous driver; FB, fat body driver; G, 1% glucose treatment; PLP, 1 mM PLP treatment; untr., untreated; wt, wild‐type. Significant difference in the Student's *t*‐test with *p < 0.05, **p < 0.01, and ***p < 0.001, respectively.

Vitamin B6 is an antioxidant molecule able to counteract AGEs and ROS (Merigliano, Mascolo, La Torre, et al., 2018). We, therefore, tested whether PLP, the biologically active form of vitamin B6, was able to rescue AGEs and ROS in *PI3K92E* ^*RNAi*^ larvae. As shown in Figure 4a,b, we found that PLP‐fed *PI3K92E* ^*RNAi*^ larvae exhibited normal levels of both AGEs and ROS. In addition, PLP rescued CABs in Dp110 depleted cells, also when brains were pretreated with glucose (Figure 4c).

On the other hand, feeding *PI3K92E* ^*RNAi*^ larvae with the PLP inhibitor 4DP enhanced CAB frequency up to 64% (vs. 20% in 4DP‐treated controls) (Figure 4d,e), thus emphasizing the protective role exerted by vitamin B6 in genome integrity maintenance in diabetes (Contestabile et al., 2020). Taken together these results reveal that, in *Drosophila*, vitamin B6 is able to reduce oxidative stress and its consequences caused by the impaired functionality of PI3K.

### 3.5 PLP rescues diabetic traits caused by Dp110 depletion

**Figure 5:**
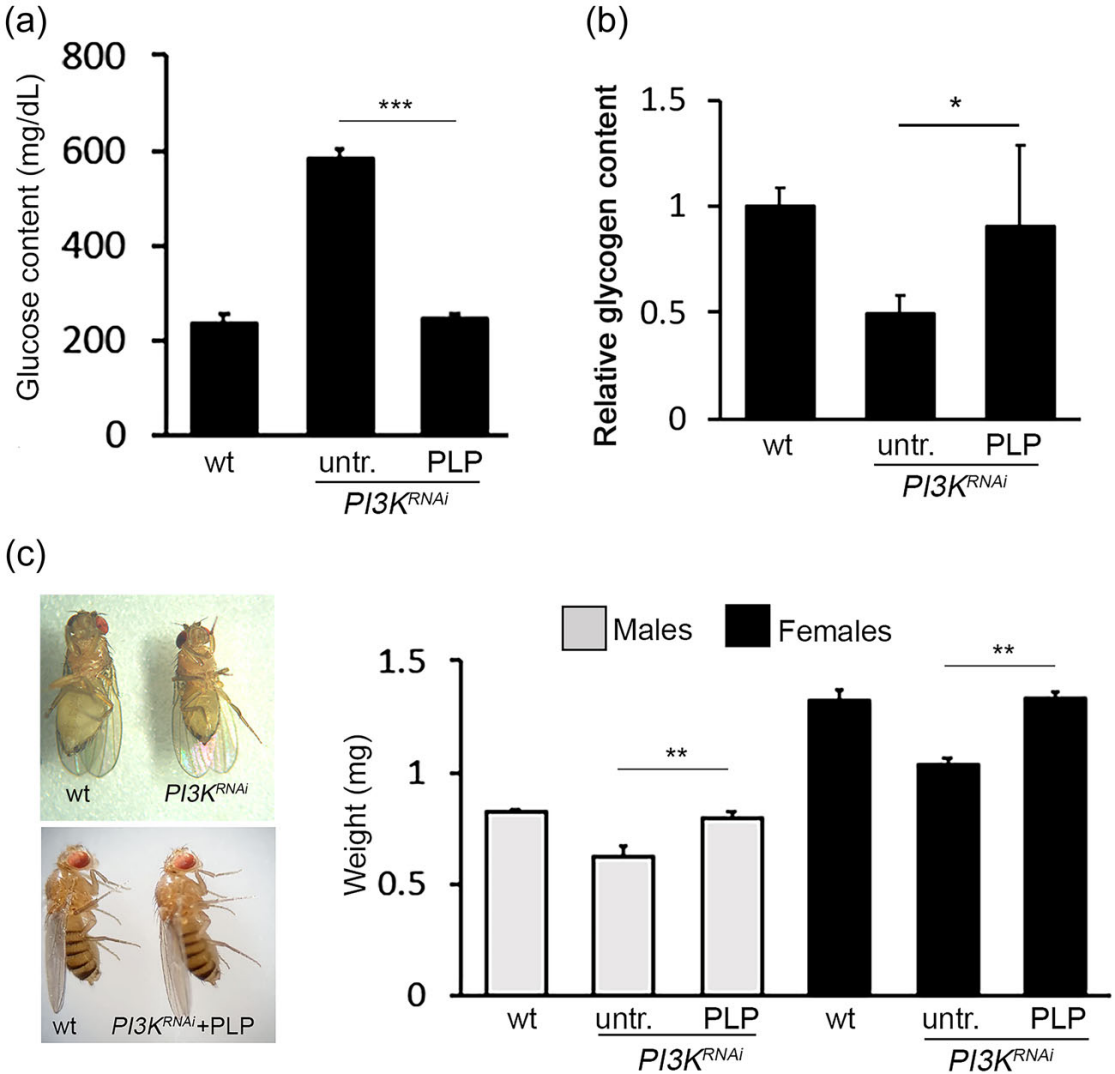
Pyridoxal 5′‐phosphate (PLP) rescues diabetic phenotypes. (a and b) Hemolymph glucose content and glycogen concentration in *PI3K* ^*RNAi*^ larvae untreated or 1 mM PLP‐treated. Columns are the means of five independent sample measurements ±*SEM*. (c) Body size rescue in PLP‐fed *PI3K* ^*RNAi*^ flies. (d) Weight measurement. Each column represents the mean weight ± *SEM* of single flies from three different experiments. PLP, 1mM PLP treatment; untr., untreated; wt, wild‐type. Significant difference in the Student's *t*‐test with *p < 0.05, **p < 0.01, and ***p < 0.001, respectively.

Mounting evidence associates vitamin B6 with diabetes, indicating that its deficiency can induce insulin resistance, while its supplementation can restore normal blood glucose levels (Abdullah et al., 2019; Fields et al., 2021; Kim et al., 2018). However, underlying mechanisms are still unknown. We thus investigated whether growing *PI3K92E* ^*RNAi*^ larvae in a medium supplemented with 1 mM PLP could suppress diabetic phenotypes. As shown in Figure 5a–d, PLP treatment rescued hyperglycemia, body size, and glycogen synthesis.

Altogether our results suggest that vitamin B6 treatment is effective in rescuing diabetes induced by PI3K depletion, possibly by enhancing insulin sensitivity.

## 4 DISCUSSION

Besides its well‐known importance in genetic research, *Drosophila melanogaster* has recently emerged as a powerful tool also in studies concerning metabolism, given that flies share with humans the majority of metabolic pathways (Das & Dobens, 2015). Fly models of type 1 diabetes and T2D have been successfully employed to better define the molecular basis of pathophysiological traits of these diseases (Liguori et al., 2021). Although human T2D is a polygenic disorder, one of the exploited strategies to obtain T2D fly models is to generate flies carrying mutations in genes belonging to the insulin pathway. This procedure allows studying all the insulin resistance hallmarks—which are the basis of human T2D—in a context where the genetic nature of the defect is known and both genetic and environmental background can be rigorously controlled (Álvarez‐Rendón et al., 2018).

In line with the functional conservation of the human insulin pathway in flies (Inoue et al., 2018), here we showed that depleting Dp110, the catalytic subunit of *Drosophila* PI3K, increases hemolymph glucose content, decreases glycogen content, and reduces the adult body size. Reduced body size is a hallmark of diabetes in *Drosophila*, because the insulin signaling pathway serves both insulin‐ and IGF‐like functions in flies (Baker & Thummel, 2007; Musselman et al., 2011). However, it has not been clarified if reduced size depends on increased apoptosis, reduced growth, or both (Böhni et al., 1999; Chen et al., 1996; Leevers et al., 1996; Weinkove et al., 1999). It has been shown that triggering the expression of dominant‐negative or constitutively active variants of Dp110‐PI3K in the developing wing and eye resulted in reduced or increased cell number and cell size, respectively (Leevers et al., 1996). Furthermore, the inhibition of Dp110 activity reduces the increase of cell number in the imaginal discs, suggesting that Dp110 normally promotes cell division and/or cell survival (Weinkove et al., 1999).

Most interestingly, we found that fat body‐specific Dp110 depletion gives rise to CABs induced by hyperglycemia. We exclude that CABs can be produced by a direct effect of *PI3K92E* silencing on DNA repair because no CABs were observed in brains in which *PI3K* was specifically silenced in this tissue (*elav‐Gal4*>*PI3K* ^*RNAi*^). Furthermore, we showed that CABs are exacerbated by glucose treatment and are accompanied by the accumulation of AGEs and ROS; remarkably, the antioxidant ALA rescues both AGEs and CABs. We can envisage a scenario in which increased circulating glucose levels, resulting from impaired PI3K function in the fat body, may favor an increased glucose uptake in neuroblasts. Thus, high glucose levels in these cells would trigger nonenzymatic glycation reactions leading to AGE production. AGEs are, in turn, responsible for ROS production during metabolism (Nowotny et al., 2015) and ROS for inducing DNA double‐strand breaks, that, if not properly repaired, lead to CABs (Kasparek & Humphrey, 2011). In addition, given that hyperglycemia can compromise DNA repair efficiency (Zhong et al., 2018), we also suppose that chromosomes of Dp110‐depleted cells may be more prone to DNA damage.

The correlation between impaired insulin signaling and CABs assumes high relevance in light of the association of T2D with an increased risk of cancer (Vigneri et al., 2009); our results, therefore, reinforce the hypothesis that one of the mechanisms by which hyperglycemia impacts on cancer is DNA damage (Deo et al., 2021). Our findings further suggest that genomic instability due to PI3K depletion should be considered an important side effect in anticancer therapies based on PI3K inhibitors, along with hyperinsulinemia and hyperglycemia (Nunnery & Mayer, 2019). Thus, identifying therapeutic, as well as dietary approaches able to minimize these effects should be of paramount importance.

Based on the evidence that vitamin B6 is an antioxidant molecule beneficial to diabetes (Merigliano, Mascolo, Burla, et al., 2018), we tested the effects of PLP supplementation in *PI3K92E* ^*RNAi*^ individuals, obtaining a complete rescue of hyperglycemia, glycogen content, AGEs, ROS, and CABs. The rescue of the oxidative stress is probably due to vitamin B6 capability of reacting against the peroxyl radicals (Kannan & Jain, 2004) and also of counteracting AGE formation by sequestering the 3‐deoxyglucoson, generated during AGE metabolism (Nakamura & Niwa, 2005). Regarding the rescue of diabetic phenotypes, we do not have an explanation, but we can envisage that PLP may potentiate the activity of the residual *PI3K* function in *PI3K* ^*RNAi*^ cells to increase insulin sensitivity. This hypothesis fits well with the finding that vitamin B6 supplementation is able to enhance insulin sensitivity, by increasing AKT phosphorylation and the expression of AKT downstream genes, in insulin‐resistant mice (Liu et al., 2016).

In conclusion, our data show that reducing PI3K activity in *Drosophila* results in hyperglycemia, reduced glycogen content, accumulation of AGEs, oxidative stress, and DNA damage; the latest aspect should be considered as a complication of the anticancer therapies based on PI3K inhibitors. Furthermore, our results reinforce the evidence that vitamin B6 treatment is a promising route to mitigate diabetes and its complications.

## AUTHOR CONTRIBUTIONS

Elisa Mascolo, Ludovica Schiano, Eleonora Gnocchini, and Eleonora Pilesi performed the experiments. Francesco Liguori, Angela Tramonti, and Chiara Merigliano performed the experiments and contributed to data analysis. Cinzia Volontè, Martino L. Di Salvo, and Roberto Contestabile contributed to data analysis and interpretation. Fiammetta Vernì conceived the study, designed the experiments, and wrote the manuscript. All authors reviewed the manuscript.

## CONFLICT OF INTEREST

The authors declare no conflict of interest.

## Supporting information

Supporting information.

## Data Availability

All data generated or analyzed during this study are included in this paper and its supporting information files.
