# Supplementary material for: Vitamin B6 rescues insulin resistance and glucose‐induced DNA damage caused by reduced activity of Drosophila PI3K
Source: J Cell Physiol. 2022 Jun 9;237(9):3578–86. doi: 10.1002/jcp.30812 (PMC9545242; doi:10.1002/jcp.30812)
Supplement: Supplementary file 1 — Supporting information. [file JCP-237-3578-s001.docx]

**SUPPORTING INFORMATION**

**
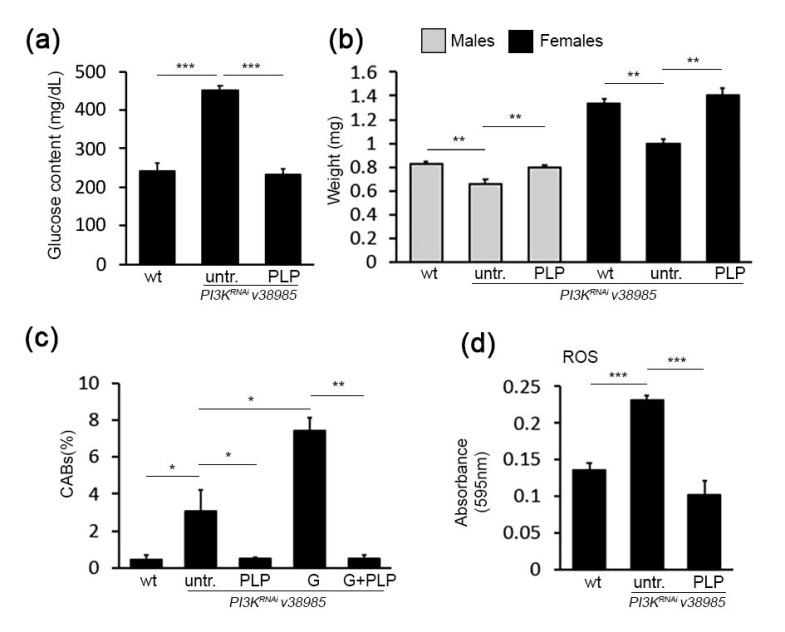
**

**FIGURE S1**

**Phenotypes induced by the inactivation of Dp110 subunit in the *PI3K92E^v38985^*RNAi line**. (a) Glucose content in larval hemolymph of *PI3K^RNAi^ v38985* larvae untreated or treated with 1 mM PLP. Columns are the mean of five independent sample measurements ± SEM. (b) Body weight (mg) measurement of *PI3K^RNAi^ v38985* flies untreated or 1 mM PLP treated. Each column represents the mean weight ± SEM of single flies. (c) CAB frequency in *PI3K^RNAi^ v38985* neuroblasts – untreated or 1% glucose treated - from larvae grown on a standard medium or 1 mM PLP supplemented medium. Each column represents the mean value ± SEM obtained by scoring at least 800 cells for each condition. (d) ROS quantification using nitroblue tetrazolium (NBT) assay in hemolymph of wild-type, untreated and 1mM PLP fed *PI3K^RNAi^ v38985* larvae. Columns represent a mean value ± SEM of three different experiments. wt: wild-type; untr.: untreated; PLP: 1mM PLP treatment; G: 1% glucose treatment. *, **, *** Significantly different in the Student’s t test with p<0.05, p<0.01 and p<0.001, respectively.


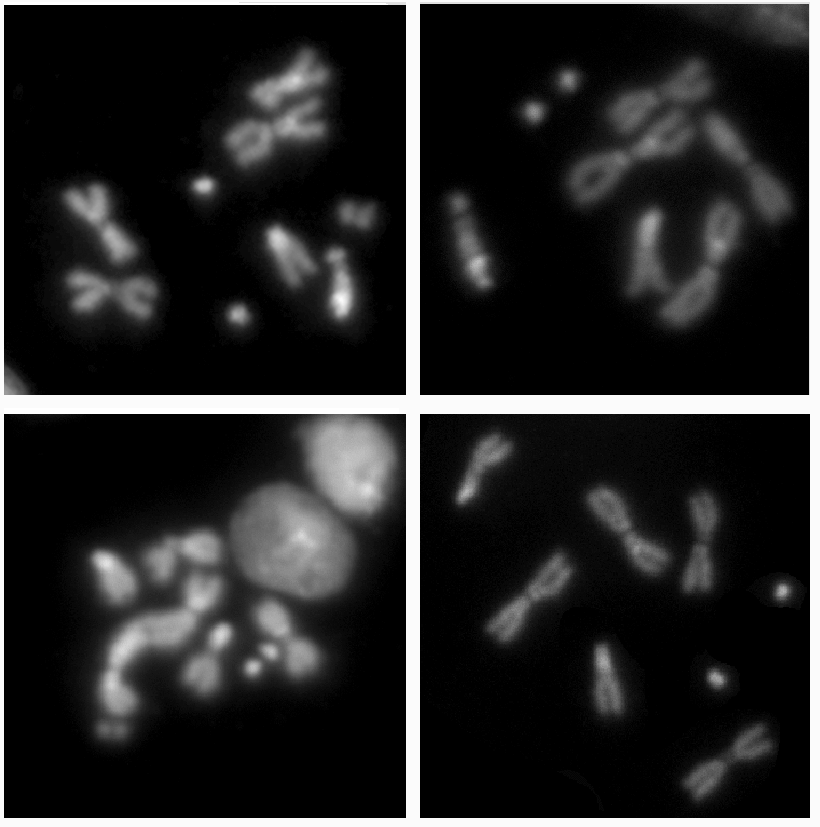


*PI3K^RNAi^*

*PI3K^RNAi^ + ALA*

*PI3K^RNAi^+G*

*PI3K^RNAi^ +G+ALA*

**FIGURE S2**

**ALA treatment rescues CABs in neuroblasts from *PI3K^RNA^*^i^ larvae**

Examples of metaphases in untreated or 1% glucose (G) treated larval brains from ALA-fed *PI3K^RNAi^* larvae. Scale bar, 5 μm.
